# Overlap syndrome of anti-aquaporin-4 positive neuromyelitis optica spectrum disorder and mixed connective tissue disease: a case report

**DOI:** 10.3389/fimmu.2025.1644259

**Published:** 2025-09-10

**Authors:** Ziyu Wang, Ling Wu, Zhihao Zhang, Chenyang Zhang, Ertao Jia, Hongling Geng

**Affiliations:** ^1^ The Fifth Clinical College of Guangzhou University of Chinese Medicine, Guangzhou, China; ^2^ Department of Rheumatology, Shenzhen Hospital, The University of Hong Kong, Shenzhen, China; ^3^ Department of Rheumatism, Guangdong Provincial Second Hospital of Traditional Chinese Medicine, Guangzhou, China; ^4^ Department of Gynecology, Guangdong Provincial Hospital of Chinese Medicine, Guangzhou, China

**Keywords:** neuromyelitis optica spectrum disorder, mixed connective tissue disease, AQP4, case report, overlap syndrome

## Abstract

Neuromyelitis optica spectrum disorder (NMOSD) is an immune-mediated inflammatory demyelinating disease affecting the optic nerve and spinal cord. NMOSD frequently coexists with other autoimmune diseases. However, its concurrence with mixed connective tissue disease (MCTD) is rather rare and often overlooked. This study reports the first case in China of aquaporin-4 immunoglobulin G (AQP4-IgG) seropositive NMOSD preceding MCTD with long-term follow-up. Between 2016 and 2024, the patient successively developed left lower limb numbness, hiccups, vomiting, facial numbness, Raynaud’s phenomenon, finger swelling, digital sclerosis, and synovitis. Acute-phase management involved pulse steroid therapy, while remission maintenance utilized azathioprine, mycophenolate mofetil, rituximab, and inebilizumab for relapse prevention. This paper presents this case and reviews other cases of NMOSD combined with MCTD, aiming to contribute to the clinical understanding and management of this rare condition.

## Introduction

Neuromyelitis optica spectrum disorder (NMOSD), formerly known as Devic’s disease, is a rare antibody-mediated central nervous system disorder. Typical manifestations include longitudinally extensive transverse myelitis, severe optic neuritis, and/or intractable vomiting and hiccups (postictal area syndrome) (1). Research indicates that aquaporin-4 immunoglobulin G (AQP4-IgG) is the causative factor of NMOSD. The pathological mechanism involves the binding of AQP4-IgG to anti-aquaporin-4 (AQP4) water channels on astrocyte foot processes, triggering complement cascade activation, resulting in astrocyte damage and subsequent secondary injury to oligodendrocytes and neurons (2).According to the revised criteria published by Wingerchuck et al., a positive AQP4 antibody with the presence of a single core clinical feature (optic neuritis, acute myelitis, or brainstem syndrome) is sufficient for diagnosis once other diseases have been ruled out (3). NMOSD may be associated with various types of autoimmune diseases. Among systemic autoimmune diseases, Sjögren’s Syndrome (SjS) and Systemic Lupus Erythematosus (SLE) were the most commonly associated with mixed connective tissue disease (MCTD). In contrast, Systemic Sclerosis (SSc) and MCTD are less common (4).

This paper reported a case of a middle-aged female with numbness of the left lower limb as the initial symptom and a positive serum AQP4-IgG, diagnosed with NMOSD. Seven years after the onset, the patient developed Raynaud’s phenomenon, swollen fingers, sclerodactyly, synovitis, alopecia, and localized cutaneous sclerosis with depigmentation on the forehead. Combined with positive anti–U1-ribonucleoprotein (RNP) antibodies and an Anti-Nuclear Antibody (ANA) titer >1:1000, the patient was ultimately diagnosed with MCTD concurrent with NMOSD. Furthermore, we also reviewed additional case reports on NMOSD coexisting with MCTD.

## Case presentation

**Figure 1:**
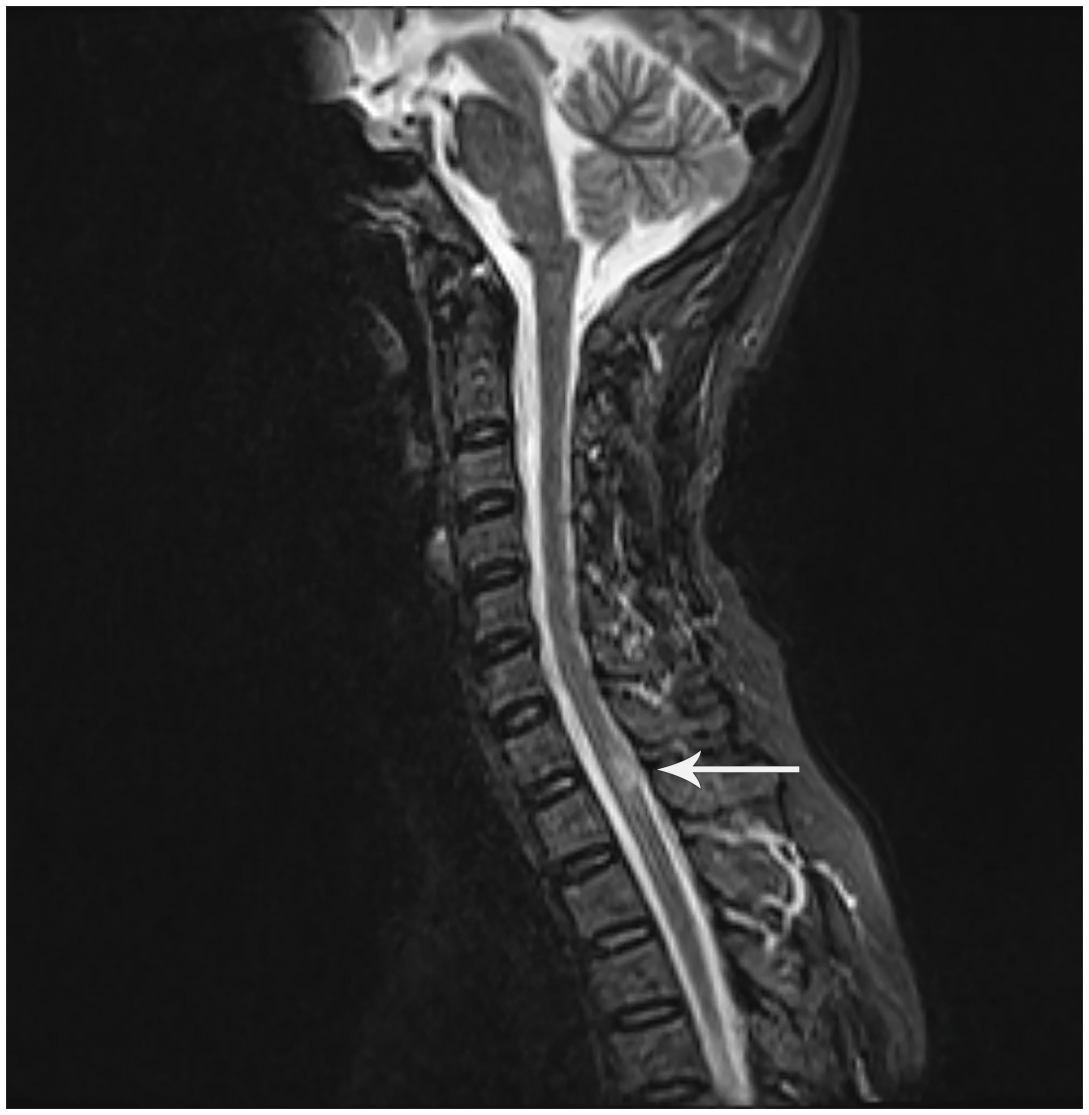
Sagittal T2-weighted MRI of the cervicothoracic spine: A hyperintense lesion at C7-T1 (white arrowhead). Although this lesion does not meet classical LETM criteria (≥3 vertebral segments), NMOSD diagnostic guidelines (Wingerchuk et al., 2015) waive the lesion length requirement for AQP4-IgG-seropositive patients. This clinically compatible lesion correlates with left lower limb numbness.

The 48-year-old female patient presented with progressive numbness in the left lower limb in June 2016, which was particularly prominent in the left foot. There was no weakness, blurred vision, dizziness, diplopia, crooked mouth, girdle sensation of the body, or disorders of urination and defecation. Among the ancillary diagnostic studies, magnetic resonance imaging (MRI) demonstrated a focal abnormal signal intensity in the spinal cord at the C7-T1 level, suggestive of a demyelinating lesion (**Figure 1**). The lumbar puncture indicated normal cerebrospinal fluid (CSF) pressure, with no significant abnormalities in the routine, biochemical, bacterial, cryptococcal, or tuberculosis examinations. Oligoclonal bands (OB) were negative; however, serum AQP4-IgG, as determined by cell-based assay (CBA), was positive with a titer of 1:100. Ultimately, she was diagnosed with NMOSD and received intravenous administration of methylprednisolone (MP) 0.5 grams/day for 5 days. Subsequently, the treatment regimen was adjusted to oral prednisone (Pred) 35 mg/day and azathioprine (AZA) 25 mg/day. Due to hepatic impairment, the Pred dosage was reduced to 5 mg/day, and the AZA dosage was increased to 50 mg/day.

In April 2018, the patient experienced recurrent hiccups and vomiting, prompting consideration of a recurrence of NMOSD, which improved with intravenous MP. During the hospitalization, a follow-up MRI examination was performed. Comparative analysis with the 2016 study demonstrated no abnormalities in spinal cord signal intensity, morphological configuration, or anatomical alignment. Subsequently, rituximab (RTX) therapy was initiated for relapse prevention. The patient received two doses, achieving sustained disease stability. However, due to personal reasons, the patient was unable to continue the injections.

**Figure 2:**
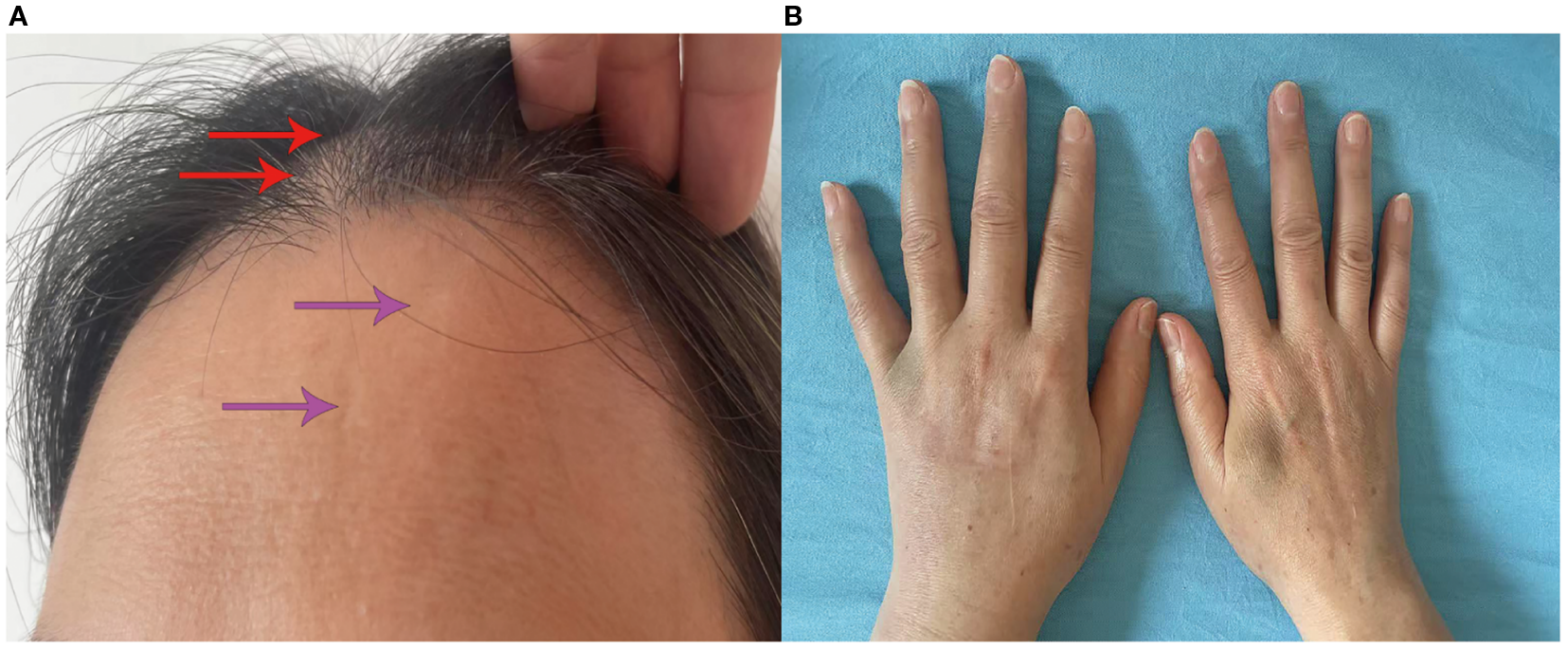
Clinical manifestations of the patient. **(A)** Alopecia (red arrowhead); localized cutaneous sclerosis with depigmentation on the forehead (purple arrowhead). **(B)** Finger swelling.

In April 2023, the patient presented with swelling of the right eyelid and face, which gradually spread to the right periauricular and external auditory canal with numbness of the right oral mucosa. The numbness eventually progressed to the left side of the face, but it was less severe than on the right side. Physical examination showed bilateral facial hyperalgesia with an onion-skin distribution, which was more prominent on the right side. Multimodal evoked potentials (somatosensory, brainstem auditory, and visual) and MRI (cranial, orbital, cervical, and thoracic) were unremarkable. Serum AQP4-IgG by CBA was positive (titer 1:100). Comprehensive laboratory investigations, including routine biochemistry, immunology, infectious serology, and hematology, showed no significant abnormalities. Given the possibility of NMOSD relapse, the patient was treated with intravenous MP at 1 g/day and maintenance therapy with mycophenolate mofetil (MMF) at 750 mg twice daily. Gabapentin was subsequently used for symptomatic treatment. Subsequently, the patient developed alopecia, Raynaud’s phenomenon, swollen fingers, frontal circumscribed scleroderma with hypopigmentation, sclerodactyly, and synovitis (**Figure 2**). The ENA profile showed anti–U1-RNP antibodies were positive and the ANA titer was >1:1000 with a speckled pattern (**Table 1**). Based on all examination results, the patient was diagnosed as NMOSD coexisting with MCTD and treated accordingly for NMOSD. The patient was successively treated with pregabalin, gabapentin, and oxcarbazepine for facial numbness, but the therapeutic effect was poor, and dizziness occurred.

**Table 1:** Summary of the immunological work up.

Immunology	Results	Reference range
ANA	> 1:1000(speckled pattern)	< 1:100
Anti–U1-RNP	> 1:1600	< 1:100
Anti-Sm	–	–
Anti-SSA	–	–
Anti-SSB	–	–
Anti-Ro-52	–	–
Anti–Scl-70	–	–
Anti-Jo-1	–	–
Anti-CENP-B	–	–
Anti-dsDNA	–	–
Anti-Nucleosome	–	–
Anti-Histone	–	–
Anti-Rib.P-Prot	–	–
C3	0.59	0.90–1.80 (g/L)
C4	0.04	0.10–0.40 (g/L)
IgM	3.24	0.40–2.30 (g/L)
IgA	6.10	0.70–4.00 (g/L)
IgG	24.13	7.00–16.00 (g/L)
ESR	28.00	< 15.00 (mm/h)

ANA, Anti-Nuclear Antibody; Anti–U1-RNP, Anti–U1-ribonucleoprotein antibodies; Sm, Smith; SSA, Sjögren’s Syndrome A; SSB, Sjögren’s Syndrome B; Scl-70, Scleroderma-70; Jo-1, Histidyl-tRNA Synthetase; CENP-B, Centromere protein B; ds-DNA, double-stranded deoxyribonucleic acid; Rib.P-Pro, Ribosomal P protein; C3, Complement 3; C4, Complement 4; IgM, Immunoglobulin M; IgA, Immunoglobulin A; IgG, Immunoglobulin G; ESR, Erythrocyte Sedimentation Rate; -, negative.

In 2024, the patient started treatment with inebilizumab (IBZ) and has received three injections so far. The patient still has a sense of numbness, tightness, and discomfort mainly on the right side of the face, accompanied by stiffness and swelling of the fingers of both hands and tenderness at the proximal interphalangeal joint of the right middle finger.

## Discussion

The case reported here involves a 48-year-old female patient with a 9-year history of NMOSD, and the AQP4 antibody was positive. Throughout treatment, the patient experienced multiple relapses of NMOSD, presenting with symptoms such as numbness in the left lower limb, hiccups, and numbness on the right side of the face. Subsequently, the patient also developed symptoms of Raynaud’s phenomenon, finger swelling, alopecia, localized cutaneous sclerosis with depigmentation on the forehead, sclerodactyly, and synovitis. Considering the positive anti–U1-RNP antibodies and an ANA titer exceeding 1:1000, the patient was diagnosed with MCTD. Following immunotherapy, the patient achieved clinical stability with only residual facial numbness and finger swelling persisting.

**Figure 3:**
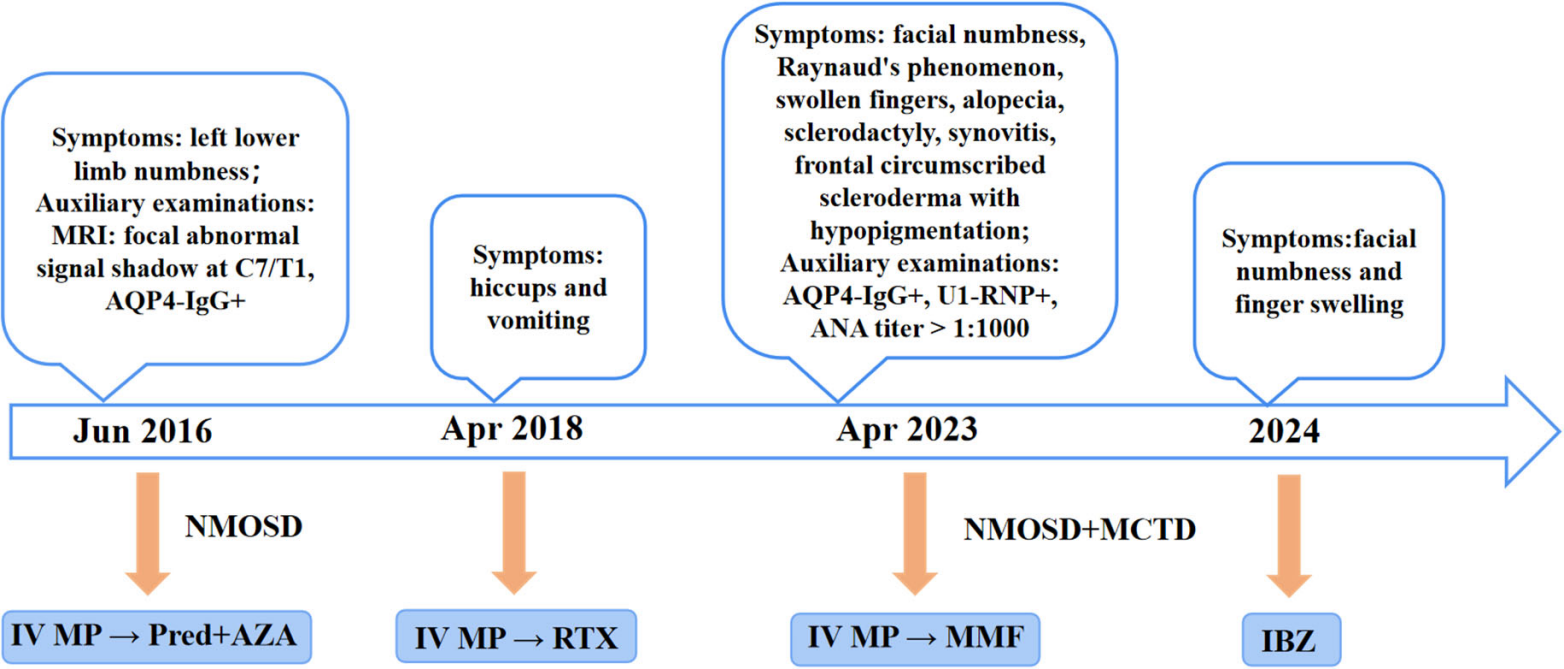
The treatment regimen for the patient, from the onset of the condition until the end of the follow-up period, is shown at different time points. IV, Intravenous; MP, Methylprednisolone; Pred, Prednisone; AZA, Azathioprine; RTX, Rituximab; MMF, Mycophenolate mofetil; IBZ, Inebilizumab.

Management of NMOSD involves acute-phase treatment and disease-modifying therapy (DMT) during remission. This case details the patient’s immunotherapy course from 2016 to 2024 (**Figure 3**). In June 2016, the patient experienced the first acute attack of NMOSD. In accordance with the criteria, high-dose intravenous MP was administered, followed by oral AZA combined with low-dose Pred for relapse prevention. In April 2018, NMOSD relapsed. After alleviation of acute symptoms, the patient was switched to RTX as DMT. Previous studies have confirmed that RTX is effective in AQP4-IgG-positive NMOSD (5). However, RTX is used off-label in China, and the patient discontinued RTX due to excessive long-term financial burden. The patient relapsed again in April 2023. Studies have shown that MMF may be a favorable therapeutic option for patients with relapse or adverse reactions during AZA treatment (6). Considering the patient’s financial status and the suboptimal efficacy of previous AZA treatment, MMF was prescribed as DMT. IBZ, a first-line maintenance treatment, has been included in China’s national medical insurance. The N-MOmentum study demonstrated that switching from RTX to IBZ results in further reduction in relapse risk (7). After comprehensive consideration, the patient was switched to IBZ in 2024. Following the third dose, the patient’s condition was stable, with only mild residual facial numbness and finger swelling. Regular follow-up assessments every 3 months showed that the patient’s symptoms continued to improve without further relapses. When formulating immunotherapy regimens, clinicians must integrate disease activity, treatment frequency, reimbursement policies, and financial constraints. This approach enhances patient compliance, facilitates long-term standardized management, and reduces the risk of disease recurrence.

Although case reports of connective tissue diseases (CTD) associated with NMOSD have been documented in the literature (8–11), those of MCTD coexisting with NMOSD remain relatively rare. Additionally, we systematically reviewed all published case reports of NMOSD combined with MCTD (**Table 2**, see Additional file 1).

**Table 2:** Systematic review of cases with neuromyelitis optica spectrum disorder complicated by mixed connective tissue disease.

Study	Gender	Ethnicity	Primary Disease and the age of onset	Secondary Disease and the age of onset	Main Symptoms	Auxiliary examinations	Treatment and Outcome
Parperis	Female	African American	MCTD, 42	NMOSD, 44	- Raynaud phenomenon, bilateral hand swelling, sclerodactyly - Progressive bilateral vision loss and intermittent episodes of paresthesias in the left forearm and hand	- Brain MRI: one area of abnormal enhancement in the posterior part of both optic nerves - Spinal MRI: diffuse spinal cord signal abnormalities from C6 to C7 - Anti-RNP+, NMO-IgG+	- Treatment: Acute: IV MP (1g/day for 3 days) → oral Pred 60mg/d + PE × 5 Maintenance: AZA + Pred - Visual acuity improved
Silva et al. (12)	Female	Brazilian	MCTD, 10	NMO, NA (April 2016)	- Raynaud phenomenon,synovitis and myositis - Burning pain in the foot sole region, pruritus, bilateral paresthesia,visual turbidity, urinary retention	- Spine MRI: Dorsal T2 hyperintensity - Orbital MRI: Bilateral intraconal optic nerve signal abnormalities (T1-T2) - Anti-RNP+, AQP4-IgG+	- Treatment: IV MP (1g/d for 5 days) → PE × 7 → IV CTX (750mg/m² monthly) NMO relapsed, IV steroids and PE → RTX (2g every 6 months) + MMF 1g/day Neurological crisis, IV MP → RTX + AZA - Symptomatic alleviation
	Female	Brazilian	MCTD, 14	NMO, 23	- photosensitivity, poliarthritis, oral ulcer, leukopenia, Raynaud phenomenon, distal dysphagia and pulmonary interstitial disease - paresthesia and symmetrical hypoesthesia of lower limbs	- Cervicodorsal spine and skull MRI showed changes - Cranial nervesshowed absence of acuity deficit, atrophy of right optic papila - Anti-RNP+, AQP4-IgG+	- Treatment: IV MP (1g/day for 3 days) → PE → CTX (750mg/m² monthly) NMO relapsed, MP → PE → CTX (1g/ month for 6 months) → RTX (2g every 6 months) and MMF (3g/day) - Disease controlled, bilateral visual acuity without luminous perception.
Polilli et al. (14)	Male	Caucasian	NA,58	NA,58	- Raynaud’s phenomenon and arthralgia often associated with morning stiffness - Unsteady gait, paraesthesia of the lower limbs, and pain in the left lumbar area of the spine	- Spine MRI: long extensive cervico-dorsal myelitis (C3–D12) - Chest CT: ground-glass opacities, interlobular septal thickening - Anti-RNP+, anti-SSA+, AQP4-IgG+	- Treatment: Acute: IV MP (1 g/day for 5 days) →oral Pred 50 mg/day Maintenance: RTX (1 g twice over a 2-week interval, and after 1 g every 6 months) - Walked normally without pain
Our case	Female	Asian	NMOSD,40	MCTD,47	- 2016: Left lower limb numbness - 2018: Recurrent hiccups and vomiting - 2023: Facial numbness, Raynaud's phenomenon, swollen fingers, alopecia, sclerodactyly, synovitis, frontal circumscribed scleroderma with hypopigmentation	- Spinal MRI: focal abnormal signal shadow at C7/T1 level - AQP4-IgG+, U1-RNP+, ANA titer > 1:1000	- Treatment: 2016: IV MP (0.5g/day for 5 days) → oral Pred 35mg/d + AZA 25mg qd 2018: IV MP → RTX 2023: IV MP 1g/d → oral MMF 750mg bid + GBP 2024: IV IBZ - Partial symptom control, but facial numbness and finger swelling persisted

MRI, Magnetic resonance imaging; C6, Cervical Vertebrae 6; T2, Thoracic Vertebra 2; MP, Methylprednisolone; IV, Intravenous; Pred, Prednisone; PE, Plasma Exchange; AZA, Azathioprine; CTX, Cyclophosphamide; RTX, Rituximab; MMF, Mycophenolate mofetil; PIP, Proximal interphalangeal; GBP, Gabapentin; IBZ, Inebilizumab; NA, Not Available.

Silva et al. (12) reported two cases of Brazilian female patients who both presented with AQP4 antibody-positive NMOSD on the basis of MCTD. However, the clinical manifestations of the two cases differed. Case 1 was a 19-year-old patient with a 9-year history of MCTD. After experiencing viral prodromal symptoms, the patient developed symptoms such as sensory abnormalities, blurred vision, urinary retention, and muscle weakness. During immunotherapy, the patient’s neurological and visual symptoms showed periodic relief. Nevertheless, the condition relapsed after the third administration of cyclophosphamide. Subsequently, the treatment was discontinued due to an allergic reaction to MMF, leading to another relapse. Fortunately, the patient’s condition improved after subsequent treatment. Case 2 was a 29-year-old patient with a 15-year history of MCTD. After presenting with acute lower limb sensory abnormalities, symmetrical sensory loss, headache, and urinary retention, the patient was diagnosed with NMOSD based on examination results. During the treatment period, the patient discontinued treatment due to pregnancy. But unfortunately, the fetus was lost at the sixth month of gestation, and NMOSD relapsed, resulting in bilateral total amaurosis and lower limb sensory abnormalities. Regrettably, despite subsequent treatment, the patient’s vision did not recover, and the optic nerves were completely atrophic.

Parperis (13) reported a case of a 44-year-old African–American female patient with a 2-year history of MCTD. The patient had previously received treatment with prednisone but failed to continue regular treatment thereafter. The patient’s visual acuity gradually declined, with the left eye being more severely affected than the right, accompanied by intermittent paresthesia in the left upper limb and hand. MRI revealed abnormal lesions in the bilateral optic nerves and diffuse abnormal signals in the C6–C7 segments. In conjunction with positive serum NMO antibody (NMO-IgG) results, the patient was diagnosed with neuromyelitis optica (NMO). After immunotherapy, the patient’s visual acuity in both eyes improved. However, following discharge, the visual acuity of the left eye continued to deteriorate, and then prophylactic treatment with AZA and Pred was initiated.

Polilli et al. (14) reported a 58-year-old Caucasian man who presented to the Emergency Department with low back pain and inability to walk. He previously manifested repeated episodes of Raynaud’s phenomenon and arthralgia. Neurological examination revealed pyramidal signs with asymmetric and progressive paraparesis associated with hypoesthesia and bladder dysfunction. Spinal MRI revealed the presence of a long, extensive cervico-dorsal myelitis. Laboratory testing showed positivity for anti–U1-RNP, anti-Sjögren’s Syndrome A (SSA), and AQP4-IgG antibodies. Symptoms resolved following high-dose MP therapy. Subsequent RTX therapy was administered, enabling the patient to achieve normal ambulation, with the latest cervico-dorsal spine MRI demonstrating negative findings.

The coexistence of NMOSD and CTD is well recognized (15). However, the exact relationship between NMOSD and rheumatic diseases has not yet been fully elucidated. Research has revealed that the coexistence of NMOSD and CTD is more prevalent in female rheumatology patients diagnosed with SjS who present with neurological symptoms, as well as in patients with neurological diseases suspected of having SjS. Additionally, NMOSD was found to have a lower incidence in SLE patients while being exceedingly rare in MCTD patients (16).

At present, there is no research that clearly defines the sequence of onset between NMOSD and MCTD/CTD. CTD may occur prior to NMOSD, or NMOSD may precede CTD, or both may occur simultaneously (8–11, 17, 18). To date, only a limited number of cases with NMOSD coexisting with MCTD have been documented. A review of these cases reveals that Silva et al. And Parperisal. reported cases where MCTD served as the initial disease. Polilli et al. described a case with simultaneous occurrence of NMOSD and MCTD, although the chronological relationship between these disorders remains debated. Notably, the patient in the current report had a history of recurrent Raynaud’s phenomenon and arthralgia, often with morning stiffness, and the spinal MRI at presentation showed spinal cord abnormalities; however, pre-diagnostic clinical details were unavailable. In contrast, this study reports the first case in China of AQP4-IgG-seropositive NMOSD preceding MCTD, with a long-term follow-up period from 2016 to 2024.

The pathogenesis of NMOSD is complex. Genetic and environmental factors play crucial roles in the occurrence and development of this disease (19). In 2004, Lennon and colleagues first discovered NMO-IgG and the following year confirmed its target as AQP4 on astrocytes (20). AQP4-IgG is highly specific for the diagnosis of NMOSD. However, in some suspected NMOSD patients with negative AQP4-IgG test results or an unknown AQP4-IgG serological status, the diagnostic work faces significant challenges. The misapplication of the diagnostic criteria for seronegative NMOSD often leads to misdiagnosis (21). Given that some NMOSD patients may be antibody-negative, strict clinical follow-up is required for suspected cases to ensure diagnostic accuracy (22).

The spinal lesion in this case did not meet classical imaging criteria for longitudinally extensive transverse myelitis (LETM). However, diagnostic guidelines permit exemption from spinal lesion length requirements in AQP4-IgG seropositive patients (3). The temporospatial correlation between progressive left lower limb numbness and a focal demyelinating lesion at C7-T1 satisfied acute myelitis criteria. Lumbar puncture demonstrated absent OB with normal CSF parameters, providing critical laboratory evidence against multiple sclerosis and supporting NMOSD. The recurrent hiccups and vomiting in 2018, consistent with area postrema syndrome—a core NMOSD clinical phenotype—further confirmed the diagnosis.

MCTD is a rare autoimmune disorder first described by Sharp et al. in 1972, characterized by the serological hallmark of anti–U1-RNP antibodies. Specific diagnostic criteria for MCTD have not yet been established by the American College of Rheumatology or the European League Against Rheumatism. Currently, the diagnostic criteria proposed by Sharp, Alarcon-Segovia, Kahn, and Kasukawa are commonly utilized (23). Among these, the Kasukawa criteria demonstrated the maximum sensitivity (77.5%), whereas the Alarcon-Segovia and Kahn criteria showed the best specificity (24). MCTD presents an extensive range of clinical manifestations. During the disease progression, it can exhibit any typical symptoms and signs of SLE, SSc, polymyositis (PM), or rheumatoid arthritis (RA). Many patients may not meet the classification criteria for MCTD early in the disease, or they only meet the diagnostic criteria of one of these autoimmune diseases (25, 26). Therefore, the diagnosis of MCTD often poses significant challenges.

This patient met core serological criteria with positive anti–U1-RNP antibodies and high-titer ANA (1:1000). Raynaud’s phenomenon and swollen fingers were present, while digital sclerosis and synovitis represented key clinical features. Based on the Kasukawa criteria, mixed connective tissue disease (MCTD) was diagnosed. Key differentiating factors included, versus SSc, the absence of anti–Scl-70/anticentromere antibodies and limited (non-diffuse) skin sclerosis; versus systemic SLE, the absence of malar rash, proteinuria, or anti-dsDNA/anti-Sm antibodies, with non-specific alopecia; and versus SjS, the absence of sicca symptoms or anti-SSA/SSB antibodies. The hallmark features confirming MCTD were high-titer anti–U1-RNP antibodies and overlapping clinical manifestations.

NMOSD is characterized by recurrence and disability, and the accumulation of disability is mainly driven by acute clinical relapses (27, 28). Kaplan-Meier survival analysis revealed that patients with NMOSD combined with CTD had a significantly earlier time to first relapse compared to those without CTD. Except for recurrence events, patients with initial NMOSD accompanied by CTD are similar to those without CTD in terms of demographic data and clinical features (29). Meanwhile, research has indicated that NMOSD patients with CTD have longer segments of spinal cord injury compared to those without CTD (30). Notably, these cases generally exhibit relapsing tendencies and varying degrees of disability. Particularly, this 29-year-old Brazilian female patient interrupted her treatment due to pregnancy, which eventually led to irreversible optic nervous system disability, and this undoubtedly had a severe impact on her daily life. Other cases lack follow-up data, with their recurrence status and current conditions unknown. Conversely, our case, after long-term follow-up, shows stable condition and continuous improvement of symptoms.

When NMOSD coexists with MCTD, the clinical manifestations become more diverse. Rheumatologists and neurologists should fully consider the potential association between NMOSD and CTD during clinical diagnosis and treatment (15). Early identification of the presence of these two diseases is crucial for the timely initiation of immunosuppressive therapy and the prevention of systemic disability.

## Conclusions

In conclusion, this study reports the first case in China of AQP4-IgG seropositive NMOSD preceding MCTD with long-term follow-up. Additionally, a review of other cases of NMOSD combined with MCTD was conducted. However, due to the rarity of this clinical condition and the scarcity of relevant literature, it is quite challenging to comprehensively document the disease course and fully confirm the correct diagnostic and treatment approaches.

## Data Availability

The original contributions presented in the study are included in the article/supplementary material. Further inquiries can be directed to the corresponding author/s.
